# Ultrasonographic Findings in Patients with Benign and Malignant Thyroid Nodules who underwent Ultrasound Guided Fine Needle Aspiration Cytology

**DOI:** 10.3889/oamjms.2015.124

**Published:** 2015-11-26

**Authors:** Venjamin Majstorov

**Affiliations:** *Institute of Pathophysiology and Nuclear Medicine, Faculty of Medicine, Ss Cyril and Methodius University of Skopje, Skopje, Republic of Macedonia*

**Keywords:** thyroid, thyroid nodules, thyroid cancer, ultrasonography, fine-needle aspiration cytology

## Abstract

**BACKGROUND:**

Patients with thyroid nodules represent common problem in daily routine of thyroidologists as well as other medical specialties. Fortunately only small number of thyroid nodules turns out to be malignant. Ultrasound is most frequently used imaging modality in the evaluation of thyroid nodules and certain ultrasonographic features are associated with greater risk for malignancy.

**AIM:**

The aim of our study was to evaluate the diagnostic performance of various ultrasonographic findings regarding thyroid malignancy.

**METHODS:**

Between September 2012 and August 2013 a total of 592 patients with 694 nodules were included in the present study. They were evaluated for thyroid nodules as a part of routine work up at outpatient’s unit of Institute of Pathophysiology and Nuclear Medicine, Medical Faculty, UKIM Skopje. In all patients thyroid ultrasound and fine needle aspiration cytology (FNAC) were performed. Surgically were removed 84 nodules and ultrasonography and cytology data were compared to histology results.

**RESULTS:**

From all examined ultrasonographic features, significant association with malignancy has been found for hypoechogenecity, marked central vascularisation, ultrasound suspicious nodules (including at least two suspicious features) and marginal for presence of microcalcifications. Highest sensitivity was obtained for hypoechogenecity, and highest specificity for microcalcifications and marked central vascularisation.

**CONCLUSION:**

Awareness of the suspicious ultrasound features is mandatory in order to optimize diagnostic and therapeutic approach to the vast number of patients with thyroid nodules.

## Introduction

Thyroid nodules are very common finding in patients and are present in as many as 3-7% of general population, if detected by palpation and in up to 70%, if detected by ultrasound [1, 2]. Use of modern imaging modalities, such as high-resolution ultrasound, has led to immense numbers of newly diagnosed patients with thyroid nodules. Fortunately, although thyroid nodules are frequent, malignant nodules are rarely found and they represent 5-15% of all nodules in many reported series [3].

It is important to select nodules that are malignant in order to provide proper further treatment for these patients and on the other side to avoid unnecessary additional investigations in patients with benign nodules. Fine needle aspiration cytology (FNAC) has been traditionally used as a fairly accurate method for defining the underlying pathology of the thyroid nodules, especially if ultrasound guided [4, 5].

Neck ultrasound is a widely available and efficient technique for examining thyroid gland and could be useful in predicting histology outcome in nodules that have been surgically removed. There are certain ultrasound findings that have been linked with benign and malignant nodules and they could be helpful in making decision which nodules should be biopsied [6-8].

The aim of the present study was to evaluate diagnostic performance of various ultrasonographic findings in patients examined for thyroid nodules.

## Material and Methods

###  Patients

In this retrospective observational study 592 consecutive patients were included (69 males, 523 females; mean age 51.2 ± 13.9 years, range 13-85), who were examined for thyroid nodules at the outpatient’s thyroid unit of the Institute of Pathophysiology and Nuclear Medicine, Medical Faculty, UKIM Skopje, in the period between September 2012 and August 2013. Thyroid ultrasound and fine needle aspiration cytology (FNAC) under ultrasonographic guidance were performed in all patients. A total of 684 thyroid nodules were biopsied and in 501 patients one, in 90 patients two and in 1 patient three nodules were subjects of FNAC. In 78 patients with 84 nodules thyroid surgery has been performed and pathology findings were used to evaluate ultrasonographic features of the nodules and cytology results.

### Thyroid Ultrasound and ultrasound guided FNAC of the thyroid nodules

Thyroid ultrasound was performed on patients laying supine with hyperextended neck by using ultrasound with a high-resolution broadband linear array transducer (LN 12-3, Philips HD6 machine). Size of the thyroid nodules (longest diameter), their echogenicity (hypoechoic, isoechoic or hyperechoic), composition (solid, mixed or cystic), margins (clear or blurred), presence of calcifications and vascularisation (no/peripheral vascularisation, limited central or marked central vascularisation assessed visually by Color/Power Doppler) were all evaluated and recorded. Ultrasound findings were considered suspicious when at least two suspicious finding were present, such as hypoechogenecity, solid composition, blurred margins, presence of microcalcifications and/or marked central vascularisation.

Enlargement of the nodules was reported by patients or found on ultrasound examination during the follow up and defined by at least 20% increase in one dimension.

Fine needle aspiration was carried out under ultrasonographic guidance by a 21G needle with at least two aspirations per nodule. Cameco syringe pistol with 20ml syringe served for aspiration of the material for cytological examination. Smears for cytology readings were obtained when contents of needles were expelled onto glass slides and smeared with a second slide. Two types of slides were done for each lesion: one fixed in 95% ethanol and Papanicolaou stained, and other air dried and May Grunwald-Giemsa stained. Cytology findings were reported by an experienced cytopathologist. Results were expressed as: 1) benign-presence of normal thyrocytes, 2) malignant-presence of cells with malignant cytological characteristics, 3) suspicious for malignancy, 4) indeterminate-in favour of follicular lesion, and 5) non adequate-only blood cells, and no presence of thyrocytes.

### Thyroid scan

In the present study in 329 (48.1%) of examined patients thyroid Tc-99m pertechnetate scan was done. Patients were imaged 20-30 minutes after intravenous application of 185MBq Tc-99m pertechnetate. Scan results were expressed as: 1) “cold”-low/absent activity in the nodule compared to the surrounding tissue, 2) indeterminate-activity in the nodule equal to the surrounding tissue and 3) “hot”-activity only in the nodule.

### Data Analysis

Continuous variables were expressed as means ± SD. Differences between means were compared with t test. Categorical variables were expressed as numbers (percents) and compared with Chi² test. Multivariate analysis was used to evaluate relationship between ultrasonographic findings and histology. P value < 0.05 was considered statistically significant. Diagnostic performance of FNAC and ultrasonographic findings were evaluated by comparing the results to histology findings.

## Results

Clinical data and ultrasound characteristics of all nodules are presented in Table 1. Most frequently found ultrasonographic features in our patients were: solid composition of the nodules (76.3%), isoechoic followed by hypoechoic pattern of echogenecity (43.1% and 39.6% respectively), clear nodule’s margins (93.4%), absence of calcifications (89.6%) and no or peripheral vascularisation in 61.5%. With US features suspicious for malignancy were 120 (17.5%) nodules and thyroid scan was done in 329 (48.1%) of all examined nodules.

**Table 1:** Clinical data of patients and ultrasonographic characteristics of thyroid nodules

Variables		
Patients, n	592
Age (y), range	51.2 ± 13.9	(13-85)
Thyroid nodules, n	684
Biopsied nodules per patient
One	501	(84.6%)
Two	90	(15.2%)
Three	1	(0.2%)
Gender
Male	69	(11.7%)
Female	523	(88.3%)
Longest diameter (mm)	22.9 ± 11.7	(6-70)
Enlargement	75	(11%)
US features
Composition
Solid	522	(76.3%)
Mixed	110	(16.1%)
Cystic	52	(7.6%)
Echogenicity
Anechoic	51	(7.5%)
Hypoechoic	271	(39.6%)
Isoechoic	295	(43.1%)
Hyperechoic	67	(9.8%)
Margins
Clear	639	(93.4%)
Blurred	45	(6.6%)
Calcifications
No calcifications	613	(89.6%)
Macrocalcifications	26	(3.8%)
Microcalcifications	45	(6.6%)
Vascularisation
No/peripheral	421	(61.5%)
Limited central	118	(17.3%)
Marked central	145	(21.2%)
US suspicious	120	(17.5%)
Thyroid scan
No	355	(51.9%)
Yes	329	(48.1%)
Cold	146/329	(44.4%)
Indeterminate	154/329	(46.8%)
Hot	29/329	(8.8%)

n, number; US, ultrasound.

Thyroid surgery underwent 78 patients and in 47 malignancies has been confirmed by histology. There were no significant differences in age and gender of the patients, nodule’s diameter, enlargement, ultrasonographic composition or margins of the nodule between benign and malignant nodules (Table 2). Malignant nodules were in 76.7% hypoechoic (p = 0.001, OR = 1.97, p = 0.001), more often had marked central vascularisation (20 nodules vs. 3 nodules, p = 0.001, OR = 7.4, p = 0.003) and US suspicious features were significantly more frequent in malignant nodules (28.6% vs. 55.1%, p = 0.02, OR = 3.1, p = 0.017). Relative risk for malignancy was 3.5 times higher in nodules with microcalcifications and showed marginal significance (8.6% vs. 24.5%, p = 0.08, OR = 3.5, p = 0.07).

**Table 2:** Comparaison of clinical and ultrasonographic characteristics of benign and malignant thyroid nodules

Variables	Benign	Malignant	P value
Patients, n	31	47	
Age (y),	48.2±14.2	50.3±14.6	ns
Range	(16-74)	(22-77)	
Gender (male)	6 (19.4%)	9 (19.1%)	ns
Thyroid nodules, n	35	49	
Longest diameter (mm)	30.7±13.9	26.9±13.2	ns
Enlargement	13 (37.1%)	15 (30.6%)	ns
US solid composition	28 (80%)	43 (87.8%)	ns
Hypoechogenicity	10 (28.6%)	33 (67.3%)	0.001
OR		1.97 (95% CI 1.3-3)	0.001
Blurred margins	4 (11.4%)	13 (26.5%)	ns
Microcalcifications	3 (8.6%)	12 (24.5%)	ns (0.08)
OR		3.5 (95% CI 0.9-13.4)	ns (0.07)
Marked central vascularisation	3 (8.6%)	20 (40.8%)	0.001
OR		7.4 (95% CI 1.98-27.4)	0.003
US suspicious	10 (28.6%)	27 (55.1%)	0.02
OR		3.1 (95% CI 1.2-7.7)	0.017
Cold nodule (scan)	13 (59.1%)	23 (82.1%)	ns

n, number; US, ultrasound.

Inadequate for FNAC interpretation were 8 (9.5%) nodules and in the remaining 76 nodules benign cytology was found in 34.5%, malignant and suspicious for malignancy were 22.6% and 25% respectively, and 7 (8.3%) were rendered indeter-minate and consistent with atypia of undetermined significance/follicular lesion according to Bethesda classification [9]. In 8 nodules considered benign by cyology final histology turned out to be malignant. There were no false positive results in cytology malignant results (Table 3).

**Table 3:** FNAC results of histologically evaluated thyroid nodules

FNAC result	All	Benign	Malignant
Benign	29 (34.5%)	21	8
Malignant	19 (22.6%)	0	19
Suspicious	21 (25%)	3	18
Indeterminate	7 (8.3%)	5	2
Inadequate	8 (9.5%)	6	2

FNAC-fine needle aspiration cytology.

Regarding the diagnostic performance of FNAC and ultrasonographic findings, highest sensitivity was found for FNAC, followed by hypoechogenicity (78.7% and 70.2% respectively), and highest specificity was obtained for marked central vascularisation and presence of microcalcification (both 90.3%, Table 4). Most accurate ultrasonographic findings in the present study were hypoechogenecity, followed by vascularisation and US suspicious nodules (69.2%, 61.5% and 61.5% respectively).

**Table 4:** Diagnostic performance of FNAC and US-findings

	Sensitivity	Specificity	PPV	NPV	Accuracy
FNAC	87.5%	92.5%	67.7%	67.7%	76.3%
Hypoechogenicity	70.2%	67.7%	76.7%	60%	69.2%
Microcalcifications	25.5%	90.3%	80%	43%	51.2%
Vascularisation+	42.6%	90.3%	86.9%	62.2%	61.5%
US suspicious	57.4%	67.7%	72.9%	51.2%	61.5%

FNAC, fine needle aspiration cytology; PPV, positive predictive value; NPV, negative predictive value.

In multivariate analysis with logistic regression ultrasonographic variables which showed significant or marginal assocciation with malignancy were entered and model with χ^2^ = 20 (p < 0.001) and accuracy of 76.2% was obtained. In this model independent predictors for malignancy were hypoechogenecity (p = 0.005) and marked central vascularisation of the nodules (p = 0.01, Table 5).

**Table 5:** Significance of ultrasonographic findings in predictive model for malignant thyroid nodules

		B	Wald	Sig.	Exp(B)	95% Cl for EXP(B)	
						Lower	Upper
Step 1^a^	Hypoecho.	-1.511	6.388	.011	.221	.068	.712
	US susp.	1.248	1.823	.177	3.482	.569	21.309
	margins	-1.453	2.650	.104	.234	.041	1.345
	microcalc.	-.988	1.100	.294	.372	.059	2.359
	vascul. +	-2.237	7.175	.007	.107	.021	.549
Step 2^a^	Hypoecho.	-1.556	6.977	.008	.211	.066	.BB9
	US susp.	.752	.983	.321	2.122	.480	9.389
	margins	-1.304	2.407	.121	.272	.052	1.410
	vase I.+	-2.017	7.016	.008	.133	.030	.592
Step 3^a^	Hypoecho.	-1.295	6.321	.012	.274	.100	.752
	margins	-.830	1.481	.224	.436	.114	1.660
	vascul.+	-1.745	6.219	.013	.175	.044	.688
Step 4^a^	Hypoecho.	-1.405	7.736	.005	.245	.091	.660
	vascul.+	-1.710	6.047	.014	.181	.046	.707

a variable(s) entered on step 1: hypoechogenecity, US suspicious, margins, microcalcifications, marked central vascularity

## Discussion

Several ultrasonografic features from those that have been evaluated in the present study have shown significant association with malignat thyroid nodules. Those were hypoechogenecity of the nodule, marked central vascularisation, presence of US suspicious features (at least two) and to some extent microcalcifications in the nodule. In a study which included 97 malignant nodules Hong et al. [10] reported that microcalcifications, hypoechogenecity and taller than wide shape of the nodule were the three sonographic fetures with significant relative risk for malignancy (RR = 3.115, 2.510 and 7.624 respectively).

We did not evaluated taller than wide shape since all three diameters were not always available in nodules we examined, but relative risk results for microcalcifications and hypoechogenecity in our study are well in agreement (OR = 3.5 and 1.97 respectively). Similarly, blurred margins were not significantly associated with malignancy in both studies. In other study that included 2445 thyroid nodules (1493 benign and 952 malignant) seven independent sonographic features that were associated with malignancy were taller than wide shape, an obscure border, an irregular margin, solid content, hypoechogenecity, microcalcifications and internal vascularisation [11]. In our study we have also found that marked central vascularisation was significantly associated with malignancy and had even the highest relative risk (OR = 7.4, p = 0.003). This was not in concordance with our results for blurred margins and we suppose the reason for this observation was that blurred or poorly defined margins are not equivalent finding to irregular margins, since irregular margins indicates that demarcation between the nodule and the surrounding tissue is clearly visible, which is not a case in former, but demonstrates an irregular, infiltrative or speculated course [12]. In a recently published study Seo et al. analyzed a total of 1058 nodules in 824 consecutive patients from which 236 nodules were malignant and found that in multivariate analysis hypoechogenecity, marked hypoechogenecity, speculated/microlobulated margin, solid content, taller than wide, microcalcification and macrocalcification were predictive of malignancy [13]. In the present study multivariate analysis we have also confirmed that hypoechogenecity was independent predictor of malignancy, but we could not confirm the same observation for microcalcifications.

In a recent meta-analysis fourteen studies with 5439 thyroid lesions (727 malignant) were included and diagnostic value of ultrasound markers of malignancy was evaluated [14]. Strongest predictors for malignancy were taller than wide shape, irregular margins and microcalcification (OR, 13.7, 7.2 and 7.1, respectively), while hypoechogenecity and intranodular flow were moderate predictors of malignancy (OR, 3.2 and 4.3, respectively). Reported pooled sensitivity and specificity for hypoechogenecity were 68.7% (95% CI, 58.8%-82.6%) and 60.3% (95% CI, 53.4%-68.2%), and for microcalcifications 44.1% (95% CI, 37.9%-51.3%) and 75.9% (95% CI, 70.3%-82%), respectively. When compared to our results, similar sensitivity and specificity were obtained for the former (70.2% and 67.7%, respectively), while for the latter sensitivity was lower and specificity higher (25.5% and 90.3%, respectively). We could assume that presence of microcalcification might had not always been correctly assigned and had led to different diagnostic performance of this feature.

Large meta-analysis with fifty-two observational studies and 12786 nodules was conducted to evaluate the diagnostic performance of ultrasonographic features for thyroid malignancy [3]. While sensitivity of ultrasonographic features associated with malignancy was rather low as in our study (26.7% to 63%, in our study 25.5% to 70.2%, respectively), four features showed relatively high specificity-microcalcifications, central vascularisation, irregular margins, and a taller than wide shape (87.8%, 78%, 83.1%, and 96.6%). Our results regarding specificity are in quite agreement with these figures, and we found highest specificity for microcalcifications and central vascularisation (90.3% for both).

Diagnostic performance of fine needle aspiration cytology (FNAC) in the present study was in concordance with previous reports [15]. Its sensitivity remains a problem even when experienced operator and cytopathologist are involved in the procedure. In the present study 8 patients with benign cytology had malignant histology. The role of ultrasonographic findings is therefore important in selection of nodules for biopsy and special caution is recommended in cases where ultrasound suspicious nodules yield benign cytology results. Lesions of undeterminate significance/follicular neoplasia (AUS/FLUS, Bethesda Category III) are problematic from diagnostic and therapeutic aspect. From 8 nodules with this cytologic characterization two had malignancy (25%), and the remaining six were benign. Lately Ho et al. reported malignancy rates of 26.6%-37.8% for AUS/FLUS nodules [16], which are higher than the predicted risk in the previous studies that ranged from 5-15% [16, 17].

In conclusion, ultrasonographic findings are not sensitive enough to be used with confidence in detecting thyroid malignancy, but some of them are very specific, such as microcalcifications and central vascularisation. Awareness of the suspicious ultrasound features is mandatory in order to optimize diagnostic and therapeutic approach to vast number of patients with thyroid nodules.

## References

[1] Tan GH, Gharib H (1997). Thyroid incidentalomas: management approaches to nonpalpable nodules discovered incidentally on thyroid imaging. Ann Intern Med.

[2] Guth S, Theune U, Aberle J, Galach A, Bamberger CM (2009). Very high prevalence of thyroid nodules detected by high frequence (13MHz) ultrasound examination. Eur J Clin Invest.

[3] Remonti LR, Kramer CK, Leitao CB, Pinto LC, Gross J (2015). Thyroid ultrasound features and risk of carcinoma: a systematic review and meta-analysis of observational studies. Thyroid.

[4] Ko HM, Jhu IK, Yang SH (2003). Clinicopathologic analysis of fine needle aspiration cytology of the thyroid. A review of 1,613 cases and correlation with histopathologic diagnoses. Acta Cytol.

[5] Cai XJ, Valiyaparambath N, Nixon P, Waghorn A, Giles T, Helliwell T (2006). Ultrasound-guided fine needle aspiration cytology in the diagnosis and management of thyroid nodules. Cytopathology.

[6] Wong KT, Ahuja AT (2005). Ultrasound of thyroid cancer. Cancer Imaging.

[7] Sipos JA (2009). Advances in ultrasound for the diagnosis and management of thyroid cancer. Thyroid.

[8] Coquia SF, Chu LC, Hamper UM (2014). The role of sonography in thyroid cancer. Radiol Clin North Am.

[9] Cibas ES, Ali SZ (2009). The Bethesda system for reporting thyroid cytopathology. Am J Clin Pathol.

[10] Hong YJ, Son EJ, Kim Jy (2010). Positive predictive values of sonographic features of solid thyroid nodule. Clin Imaging.

[11] Xu SY, Zhan WW, Wang WH (2015). Evaluation of thyroid nodules by a scoring and categorizing method based on sonographic features. J Ultrasound Med.

[12] Haugen BR, Alexander EK, Bible KC (2015). American Thyroid Association management guidelines for adult patients with thyroid nodules and differentiated thyroid cancer. Thyroid.

[13] Seo H, Na DG, Kim JH, Kim KW, Yoon JW (2015). Ultrasound-based risk stratification for malignancy in thyroid nodules: a four-tier categoriazation system. Eur Radiol.

[14] Wolinski K, Szkudlarek M, Szczepanek-Paulska E, Ruchala M (2014). Usefulness of different ultrasound features of malignancy in predicting the type of thyroid lesions: a meta-analysis of prospective studies. Pol Arch Med Wewn.

[15] Giovanella L, Ceriani L, Suriano S (2011). Lymph Node Thyroglobulin Measurement in Diagnosis of Neck Metastases of Differentiated Thyroid Carcinoma. J Thyroid Res.

[16] Ho AS, Sarti EE, Jain KS (2014). Malignancy rate in thyroid nodules classified as Bethesda category III (AYS/FLUS). Thyroid.

[17] Gweon HM, Son EJ, Youk JH, Kim JA (2013). Thyroid nodules with Bethesda system III cytology: can ultrasonography guide the next step?. Ann Surg Oncol.

